# The effects of antidiabetic agents on heart failure

**DOI:** 10.1007/s12471-021-01579-2

**Published:** 2021-06-07

**Authors:** M. Wijnen, E. J. J. Duschek, H. Boom, M. van Vliet

**Affiliations:** 1 Department of Internal Medicine, Reinier de Graaf Gasthuis, Delft, The Netherlands; 2 Department of Endocrinology, Reinier de Graaf Gasthuis, Delft, The Netherlands; 3 Department of Nephrology, Reinier de Graaf Gasthuis, Delft, The Netherlands; 4 Department of Cardiology, Reinier de Graaf Gasthuis, Delft, The Netherlands

**Keywords:** Heart failure, Diabetes mellitus type 2, Pharmacology

## Abstract

In the Netherlands, approximately 250,000 people are living with heart failure. About one-third of them have comorbid diabetes mellitus type 2. Until recently, the effects of antidiabetic agents on heart failure were largely unknown. This changed after an observed increased risk of heart failure and ischaemic heart disease associated with thiazolidinediones that prompted the requirement for cardiovascular outcome trials for new glucose-lowering drugs. In the past decade, three new classes of antidiabetic agents have become available (i.e. dipeptidyl peptidase‑4 inhibitors, glucagon-like peptide‑1 receptor agonists and sodium-glucose cotransporter‑2 (SGLT2) inhibitors). Although the first two classes demonstrated no beneficial effects on heart failure compared to placebo in patients with diabetes mellitus type 2, SGLT2 inhibitors significantly and consistently lowered the risk of incident and worsening heart failure. Two recent trials indicated that these favourable effects were also present in non-diabetic patients with heart failure with reduced ejection fraction, resulting in significantly lower risks of hospitalisation for heart failure and presumably also cardiovascular and all-cause mortality. SGLT2 inhibitors have been shown to be benefit on top of recommended heart failure therapy including sacubitril/valsartan and may also prove beneficial for heart failure with preserved ejection fraction. In this review, we discuss the effects of antidiabetic agents on heart failure.

## Box 1 Indications for prescribing sodium-glucose cotransporter-2 (SGLT2) inhibitors and points of concern in patients with heart failure with reduced ejection fraction (HFrEF)

- On 5 November 2020, dapagliflozin was approved for the treatment of HFrEF in diabetic and non-diabetic patients (with an estimated glomerular filtration rate ≥ 30 ml/min per 1.73 m^2^) in Europe and may consequently be prescribed for this indication.
- SGLT2 inhibitors can be prescribed to patients with HFrEF in addition to angiotensin-neprilysin inhibitors, mineralocorticoid receptor antagonists and beta-blockers.
- SGLT2 inhibitors can be prescribed to outpatients as well as inpatients (e.g. during a hospital stay for acute decompensated heart failure).
- Common side effects of SGLT2 inhibitors include genital infections and volume depletion. Doses of diuretics may have to be adjusted in patients who are prone to hypovolaemia.
- In patients using sulfonylurea derivates or insulin, consultation with an endocrinologist is recommended before prescribing SGLT2 inhibitors due to an increased risk of hypoglycaemia and euglycaemic ketoacidosis.
- It is important to communicate the prescription of SGLT2 inhibitors to the general practitioner because most patients with diabetes mellitus type 2 in the Netherlands are treated by a family doctor.

## Box 2 Dutch contribution to studies on the effects of antidiabetic agents on heart failure

- Many cardiovascular outcome trials on antidiabetic agents have included Dutch patients due to multinational collaboration involving Dutch institutions.
- Several Dutch institutions also participated in the multinational DAPA-HF and EMPEROR-Reduced trials that for the first time investigated the effects of sodium-glucose cotransporter‑2 (SGLT2) inhibitors on heart failure with reduced ejection fraction in non-diabetic patients.
- A dedicated multi-institutional study from the Netherlands was first to investigate the effects of SGLT2 inhibitors in patients with acute decompensated heart failure.
- Dutch researchers were also involved in basic and translational studies on mechanisms underlying the beneficial effects of SGLT2 inhibitors on heart failure risk.

## Introduction

In the Netherlands, approximately 250,000 people are living with heart failure [1]. The term heart failure describes the signs and symptoms of underlying heart disease that results in elevated cardiac pressures or reduced cardiac output, regardless of aetiology, and is mainly subdivided according to left ventricular ejection fraction (LVEF) into heart failure with reduced (LVEF ≤ 40%), mid-range (LVEF 41–49%) and preserved (LVEF ≥ 50%) ejection fraction (HFrEF, HFmrEF and HFpEF) [2]. Up until now, most studies on heart failure have focused on HFrEF and, to a lesser extent, HFpEF.

Diabetes mellitus type 2 is a frequent cause and comorbidity in patients with heart failure, affecting approximately one-third of the patient population [3]. Heart failure in patients with diabetes mellitus type 2 is caused by both ischaemic cardiomyopathy due to advanced atherosclerosis and diabetic cardiomyopathy [4]. A cross-sectional study from the Netherlands demonstrated that 6% and 25% of patients with diabetes mellitus type 2 aged 60 years or older suffer from HFrEF and HFpEF, respectively [5]. Prognosis of heart failure is worse in patients with compared to those without diabetes mellitus type 2. This is illustrated by a recent meta-analysis that included data from 381,275 individuals with heart failure that demonstrated a significantly increased risk of all-cause mortality (hazard ratio (HR) 1.28 (95% confidence interval (CI) 1.21–1.35)), cardiovascular mortality (HR 1.34 (95% CI 1.20–1.49)) and hospitalisation for heart failure (HR 1.35 (95% CI 1.20–1.50)) associated with diabetes mellitus type 2 [6].

Until recently, the effects of antidiabetic agents on heart failure were largely unknown. This changed after an observed increased risk of incident and worsening heart failure, as well as ischaemic heart disease associated with thiazolidinediones that prompted the requirement for cardiovascular outcome trials for new glucose-lowering drugs in 2008 [7]. In cardiovascular outcome trials, new antidiabetic agents are compared to placebo regarding cardiovascular mortality, non-fatal myocardial infarction and non-fatal ischaemic stroke. Hospitalisation for heart failure and declining kidney function are generally included as secondary endpoints in these studies. In the past decade, three classes of antidiabetic agents have been investigated in cardiovascular outcome trials, including dipeptidyl peptidase‑4 (DPP4) inhibitors, glucagon-like peptide‑1 receptor (GLP1R) agonists and sodium-glucose cotransporter‑2 (SGLT2) inhibitors.

In this review, we discuss the effects of antidiabetic agents on heart failure with a focus on new glucose-lowering drug classes.

## Methods

For this narrative review, we searched PubMed for articles on heart failure and antidiabetic agents published up to December 2020. We used ‘heart failure’, ‘diabetes mellitus type 2’, ‘metformin’, ‘sulfonylurea derivates’, ‘insulin’, ‘thiazolidinediones’, ‘dipeptidyl peptidase‑4 inhibitors’, ‘glucagon-like peptide‑1 receptor agonists’ and ‘sodium-glucose cotransporter‑2 inhibitors’ as search terms. We focused on meta-analyses, review articles and randomised controlled trials but did not exclude other studies. We checked the reference lists of selected articles for additional relevant works. We restricted our search to articles written in English or Dutch.

## Results

### The era before cardiovascular outcome trials

#### Metformin

Metformin is a biguanide that is postulated to potentiate insulin sensitivity by inhibition of mitochondrial enzymes and is generally the first-line medical treatment for diabetes mellitus type 2 [8]. It is weight-neutral, inexpensive, and does not induce hypoglycaemia. The choice of metformin as a first-line antidiabetic agent has largely relied on encouraging results on cardiovascular risk reduction observed in the UKPDS (United Kingdom Prospective Diabetes Study) [9]. Initially, metformin was contraindicated in patients with heart failure due to concerns regarding lactic acidosis. However, these concerns were refuted in 2006 [10].

The effects of metformin on heart failure were investigated in a meta-analysis by Crowley et al. that pooled data from 35,410 diabetic patients with heart failure from 11 observational studies [11]. Metformin was associated with a significantly lower all-cause mortality (HR 0.78 (95% CI 0.71–0.87)) and risk of hospitalisation for heart failure (HR 0.87 (95% CI 0.78–0.97)) compared to other glucose-lowering drugs. Cardiovascular mortality, however, was not significantly lower in patients treated with metformin (HR 0.77 (95% CI 0.53–1.12)). Unfortunately, due to insufficient data, Crowley et al. could not subdivide the results according to HFrEF and HFpEF.

A more recent meta-analysis by Halabi et al. that specifically investigated all-cause mortality in diabetic patients with heart failure did stratify results according to HFrEF and HFpEF [12]. In this meta-analysis, which pooled data of 22,469 patients from four observational studies (including three that were also included by Crowley et al.), metformin reduced all-cause mortality in patients with HFpEF (β = −2.3 (95% CI −3.3 to −1.3)) but not HFrEF (β = 0.2 (95% CI −0.2 to 0.6)).

#### Sulfonylurea derivates

Sulfonylurea derivates lower glucose by stimulating endogenous insulin production and consequently predispose to hypoglycaemia and weight gain [8]. Current evidence is inconclusive regarding the effects of sulfonylurea derivates on heart failure. In the UKPDS, 3867 patients with newly diagnosed diabetes mellitus type 2 were randomly assigned to an intensive glucose-lowering strategy with sulfonylurea derivates or insulin versus a conventional glucose-lowering strategy with diet [13]. After 10 years of follow-up, the risk of incident heart failure was not significantly different between the two treatment strategies (HR 0.91 (95% CI 0.54–1.52)). However, results from the UKPDS may not be applicable to a large proportion of the diabetic population, since only patients with low cardiovascular risk and without baseline heart failure were included.

In contrast to the neutral outcome of the UKPDS, another study demonstrated an increased risk of heart failure associated with sulfonylurea derivates. This recent retrospective population-based cohort study by Xu et al. included 15,752 patients with diabetes mellitus type 2 from the Yinzhou district in China and observed a significantly increased risk of hospitalisation for heart failure in patients treated with sulfonylurea derivate monotherapy compared to acarbose monotherapy (an alpha-glucosidase inhibitor preventing intestinal glucose absorption with neutral effects on heart failure that is not commonly prescribed in the Netherlands) (HR 1.61 (95% CI 1.14–2.27)) [14].

#### Insulin

Exogenous insulin may cause oedema by increasing sodium reabsorption in the distal tubule of the kidney [8]. This may hypothetically increase the risk of heart failure. However, evidence regarding the effects of insulin on heart failure is conflicting. The risk of incident heart failure associated with insulin was investigated in the ORIGIN (Outcome Reduction with an Initial Glargine Intervention) trial [15]. ORIGIN included 12,537 patients with either prediabetes or diabetes mellitus type 2 and found no increased risk of heart failure with basal insulin compared to placebo (HR 0.90 [95% CI 0.77–1.05]).

Although ORIGIN indicated that insulin is safe regarding the risk of incident heart failure, an observational study called CHARM (Candesartan in Heart Failure Assessment of Reduction in Mortality and Morbidity) demonstrated a significantly increased risk of all-cause mortality (HR 1.25 (95% CI 1.03–1.51)), as well as a significantly increased risk of a composite outcome of cardiovascular death and hospitalisation for heart failure (HR 2.03 (95% CI 1.80–2.29)) associated with insulin in patients with established heart failure [16]. While CHARM included both patients with HFrEF and HFpEF, results were not compared according to heart failure subtype.

#### Thiazolidinediones

Thiazolidinediones are agonists of the nuclear peroxisome proliferator-activated receptor-gamma that increase insulin sensitivity. Fluid retention is a major side effect and may significantly increase the risk of incident and worsening heart failure [8]. This is illustrated by a meta-analysis by Lago et al. that included data from seven studies and reported a relative risk of heart failure of 1.72 (95% CI 1.21–2.42) with thiazolidinediones compared to placebo [17]. Lago et al. included data from patients with prediabetes and diabetes mellitus type 2 with or without established heart failure.

## The era of cardiovascular outcome trials

### DPP4 inhibitors

DPP4 inhibitors impair the degradation of the gut hormones glucagon-like peptide‑1 and gastric inhibitory polypeptide. Thereby they mimic the incretin effect. The incretin effect is characterised by increased insulin production after oral relative to intravenous glucose challenge and is responsible for more than half of the meal-related insulin secretion in healthy individuals [18]. Incretin-mimetic antidiabetic agents only exert glucose-lowering effects after food intake and consequently do not cause hypoglycaemia. To date, five cardiovascular outcome trials have investigated DPP4 inhibitors (Tab. 1; [19–23]). A recent meta-analysis by Sinha and Ghosal that included data from four of these trials reported neutral effects of DPP4 inhibitors on hospitalisation for heart failure (OR 1.06 (95% CI 0.96–1.18)). Occurrence of cardiovascular death, myocardial infarction and ischaemic stroke were also similar between patients treated with DPP4 inhibitors and placebo [24].

**Table 1:** Risk of heart failure exacerbation in cardiovascular outcome trials on dipeptidyl peptidase‑4 inhibitors

Study	Drug	*N*	Baseline heart failure (%)	Baseline cardiovascular disease (%)	Median follow-up (years)	Heart failure hospitalisation risk (HR (95% CI))
SAVOR-TIMI 53 [19]	Saxagliptin	16,492	13	78	2.1	1.27 (1.07–1.51)
EXAMINE [20]	Alogliptin	5380	28	100	1.5	1.07 (0.79–1.46)
TECOS [21]	Sitagliptin	14,671	18	100	3.0	1.00 (0.83–1.20)
CARMELINA [22]	Linagliptin	6991	27	57	2.2	0.90 (0.74–1.08)
CAROLINA^a^ [23]	Linagliptin	6033	4	42	5.9	1.21 (0.92–1.59)

*N* number,* HR* hazard ratio,* CI* confidence interval

^a^Compared with glimepiride instead of placebo

One of the cardiovascular outcome trials on DPP4 inhibitors that was also included in the meta-analysis by Sinha and Ghosal, SAVOR-TIMI 53 (Saxagliptin Assessment of Vascular Outcomes Recorded in Patients with Diabetes Mellitus—Thrombolysis in Myocardial Infarction 53), demonstrated a significantly increased risk of hospitalisation for heart failure associated with saxagliptin compared to placebo (HR 1.27 (95% CI 1.07–1.51)) [19]. A post hoc analysis of this trial revealed a significantly higher risk of hospitalisation for heart failure in patients with a higher baseline N‑terminal pro-B-type natriuretic peptide (NT-proBNP) level and lower kidney function [25].

Because the cardiovascular outcome trials on DPP4 inhibitors included a relatively low number of patients with heart failure, the VIVIDD (Vildagliptin in Ventricular Dysfunction Diabetes) trial was conducted [26]. VIVIDD randomised 254 diabetic patients with established HFrEF (New York Heart Association (NYHA) class I to III) to treatment with vildagliptin or placebo and investigated echocardiographic changes in LVEF, and the number of hospitalisations for heart failure after 1 year of follow-up. Change in LVEF (4.95 ± 1.25% in vildagliptin vs 4.33 ± 1.23% in placebo; difference 0.62% (95% CI −2.21% to 3.44%)) and the number of hospitalisations for heart failure (13 vs 10; *P* *=* 0.55) were comparable between patients treated with vildagliptin and placebo [26].

These data indicate that the increased risk of heart failure hospitalisation is likely specific to saxagliptin and not a class effect of DPP4 inhibitors. Variation in substrate selectivity between different DPP4 inhibitors has been postulated as the underlying mechanism of variance in heart failure risk associated with various agents [27].

### GLP1R agonists

GLP1R agonists also mimic the incretin effect. In addition to their glucose-lowering potential, they stimulate weight loss and result in lower blood pressure due to afterload reduction and natriuresis [8]. To date, seven cardiovascular outcome trials have investigated GLP1R agonists (Tab. 2; [28–34]). A recent meta-analysis by Kristensen et al. that included data from all of these studies demonstrated a significant reduction in hospitalisation for heart failure in patients treated with GLP1R agonists relative to placebo (HR 0.88 (95% CI 0.82–0.94)). Cardiovascular death, myocardial infarction and ischaemic stroke were also significantly lower in patients treated with GLP1R agonists [35].

**Table 2:** Risk of heart failure exacerbation in cardiovascular outcome trials on glucagon-like peptide‑1 receptor agonists

Study	Drug	*N*	Baseline heart failure (%)	Baseline cardiovascular disease (%)	Median follow-up (years)	Heart failure hospitalisation risk (HR (95%CI))
ELIXA [28]	Lixisenatide	6068	22	100	2.1	0.96 (0.75–1.23)
LEADER [29]	Liraglutide	9340	18	81	3.8	0.87 (0.73–1.05)
SUSTAIN‑6 [30]	Semaglutide	3297	24	83	2.1	1.11 (0.77–1.61)
EXSCEL [31]	Exenatide	14,752	16	73	3.2	0.94 (0.78–1.13)
HARMONY [32]	Albiglutide	9463	20	100	1.6	0.85 (0.70–1.04)
REWIND [33]	Dulaglutide	9901	9	32	5.4	0.93 (0.77–1.12)
PIONEER‑6 [34]	Semaglutide	3182	NA	85	1.3	0.86 (0.48–1.55)

*N* number, *HR* hazard ratio,* CI* confidence interval, *NA* not available

Two small trials specifically investigated the GLP1R agonist liraglutide in patients with HFrEF [36, 37]. Both included patients with and without diabetes mellitus type 2. The first trial, called FIGHT (Functional Impact of GLP‑1 for Heart Failure Treatment), randomised 300 patients with acute decompensated heart failure to liraglutide or placebo [36]. Liraglutide did not result in a lower risk of death or hospitalisation for heart failure (HR 1.30 (95% CI 0.92–1.83)). The second study, called LIVE (Effect of Liraglutide, a Glucagon-like Peptide‑1 Analogue, on Left Ventricular Function in Stable Chronic Heart Failure Patients With and Without Diabetes), included 241 patients with stable heart failure and found no significant change in LVEF after 24 weeks of treatment with liraglutide versus placebo (mean difference −0.8% (95% CI −2.1% to 0.5%)). However, the number of cardiac events, including arrhythmia and ischaemic heart disease, was significantly higher in patients treated with liraglutide (12 vs 3; *P* < 0.05) [37]. This latter finding may be due to an adverse cardiovascular effect of an increased heart rate associated with liraglutide [38].

### SGLT2 inhibitors

SGLT2 inhibitors are antidiabetic agents that lower blood glucose by inducing glycosuria. To date, four cardiovascular outcome trials have evaluated SGLT2 inhibitors in patients with diabetes mellitus type 2 (Tab. 3; [39–42]). A recent meta-analysis by McGuire et al. that included data from all of these trials demonstrated a significant reduction in a composite outcome of hospitalisation for heart failure and cardiovascular death in patients treated with SGLT2 inhibitors compared to placebo (HR 0.78 (95% CI 0.73–0.84)). The occurrence of myocardial infarction, ischaemic stroke and declining kidney function was also significantly lower in patients treated with SGLT2 inhibitors [43]. The benefit regarding heart failure was similar in patients with or without baseline heart failure and patients with or without atherosclerotic cardiovascular disease. Unfortunately, the results could not be compared according to heart failure subtype.

**Table 3:** Risk of heart failure exacerbation in cardiovascular outcome, heart failure and kidney outcome trials on sodium-glucose cotransporter‑2 (SGLT2) inhibitors

Study	Drug	*N*	Baseline heart failure (%)	Baseline cardiovascular disease (%)	Median follow-up (years)	Heart failure hospitalisation risk (HR (95% CI))
*Cardiovascular outcome trials*						
EMPA-REG [39]	Empagliflozin	7020	10	99	3.1	0.65 (0.50–0.85)
CANVAS Program [40]	Canagliflozin	10,142	14	66	2.4	0.67 (0.52–0.87)
DECLARE-TIMI 58 [41]	Dapagliflozin	17,160	10	41	4.2	0.73 (0.61–0.88)
VERTIS CV [42]	Ertugliflozin	8246	24	76	3.0	0.70 (0.54–0.90)
*Heart failure trials*						
DAPA-HF^a^ [45]	Dapagliflozin	4744	100	56	1.5	0.70 (0.59–0.83)
EMPEROR-Reduced^b^ [46]	Empagliflozin	3730	100	52	1.3	0.70 (0.58–0.85)
SCORED [60]	Sotagliflozin^c^	10,584	31	22	1.3	0.67 (0.55–0.82)
SOLOIST-WHF [49]	Sotagliflozin^c^	1222	100	NA	0.8	0.64 (0.49–0.83)
*Kidney outcome trials*						
CREDENCE [50]	Canagliflozin	4401	15	50	2.6	0.61 (0.47–0.80)
DAPA-CKD^d^ [51]	Dapagliflozin	4304	11	37	2.4	0.71 (0.55–0.92)^e^

*N* number, *HR* hazard ratio,* CI* confidence interval, *NA* not available

^a^Included 1983 diabetic and 2761 non-diabetic patients with heart failure with reduced ejection fraction (HFrEF)

^b^Included 1856 diabetic and 1874 non-diabetic patients with HFrEF

^c^Sotagliflozin is a SGLT2 inhibitor with some anti-SGLT1 activity

^d^Included 2906 diabetic and 1398 non-diabetic patients with chronic kidney disease

^e^Composite outcome of heart failure hospitalisation and cardiovascular mortality

A secondary exploratory analysis of the CANVAS (Canagliflozin Cardiovascular Assessment Study) Program (one of the studies included in the meta-analysis by McGuire et al.), however, did compare a composite outcome of hospitalisation for heart failure and cardiovascular death according to heart failure subtype [44]. Although the risk of the composite outcome was on the borderline of being significantly lower in patients with HFrEF treated with canagliflozin compared to placebo (HR 0.69 (95% CI 0.48–1.00)), it was not for patients with HFpEF (HR 0.83 (95% CI 0.55–1.25)). While this result argues against a significant effect of canagliflozin on HFpEF, the analysis might have been underpowered to detect a true difference.

The beneficial effects of SGLT2 inhibitors on heart failure in patients with diabetes mellitus type 2 prompted the inception of the DAPA-HF (Dapagliflozin and Prevention of Adverse Outcomes in Heart Failure) and EMPEROR-Reduced (Empagliflozin Outcome Trial in Patients with Chronic Heart Failure with Reduced Ejection Fraction) trials [45, 46]. These trials were the first to demonstrate a significant beneficial effect of SGLT2 inhibitors on HFrEF in patients without diabetes mellitus. DAPA-HF included 1983 diabetic and 2761 non-diabetic patients with baseline HFrEF (NYHA class II to IV). After a median follow-up of 1.5 years, the occurrence of a composite outcome of worsening heart failure and cardiovascular death was significantly lower in patients treated with dapagliflozin compared to placebo (HR 0.74 (95% CI 0.65–0.85)) [45]. Results were similar in patients with and without diabetes mellitus type 2.

EMPEROR-Reduced included 1856 diabetic and 1874 non-diabetic patients with HFrEF (NYHA class II to IV). After 1.3 years of follow-up, a composite outcome of hospitalisation for heart failure and cardiovascular death was significantly less common in patients treated with empagliflozin relative to placebo (HR 0.75 (95% CI 0.65–0.86)). Results were comparable between diabetic and non-diabetic patients, as well as between patients treated with or without sacubitril/valsartan [46].

Although both DAPA-HF and EMPEROR-Reduced demonstrated a significant reduction in a composite outcome of cardiovascular death and hospitalisation for heart failure associated with SGLT2 inhibitors, after separation of these events, cardiovascular mortality was only significantly lowered in DAPA-HF [45, 46]. The absence of a mortality benefit in EMPEROR-Reduced may be due to a lack of power for this outcome variable. EMPEROR-Reduced included patients with a lower LVEF (mean 27% vs 31%) and higher NT-proBNP level (median 1907 vs 1437 pg/ml) compared to DAPA-HF. This resulted in a smaller study population with a similar number of events regarding hospitalisation for heart failure but a lower number of (cardiovascular) deaths in EMPEROR-Reduced compared to DAPA-HF (588 vs 549 for heart failure hospitalisation; 389 vs 500 for cardiovascular deaths; and 515 vs 605 for all deaths, respectively) [45, 46]. Importantly, a recent meta-analysis by Zannad et al. that pooled data from both DAPA-HF and EMPEROR-Reduced demonstrated that SGLT2 inhibitors significantly lowered cardiovascular mortality (HR 0.86 (95% CI 0.76–0.98)) and all-cause mortality (HR 0.87 (95% CI 0.77–0.98)) compared to placebo [47].

The aforementioned beneficial findings of SGLT2 inhibitors in HFrEF were corroborated in patients with acute decompensated heart failure in the Dutch multicentre trial EMPA-RESPONSE-AHF (Randomized, Double-blind, Placebo-controlled, Multicentre Pilot Study on the Effects of Empagliflozin on Clinical Outcomes in Patients with Acute Decompensated Heart Failure) and the multinational SOLOIST-WHF (Effect of Sotagliflozin on Cardiovascular Events in Patients with Type 2 Diabetes Post Worsening Heart Failure) trial [48, 49]. EMPA-RESPONSE-AHF randomised 80 patients with acute decompensated heart failure with or without diabetes mellitus type 2 to treatment with empagliflozin or placebo. Empagliflozin significantly decreased a composite outcome of in-hospital worsening heart failure and rehospitalisation for heart failure or death at 60 days after enrolment (10% vs 33%, *p* < 0.05) [48]. SOLOIST-WHF randomised 1222 diabetic patients hospitalised for acute decompensated heart failure to sotagliflozin or placebo. Treatment with sotagliflozin significantly reduced the composite outcome of cardiovascular death and worsening heart failure (HR 0.67 (95% CI 0.52–0.85)), irrespective of drug initiation before or after hospital discharge [49].

SGLT‑2 inhibitors have also been shown to decrease the risk of worsening kidney function in both diabetic and non-diabetic patients with chronic kidney disease [50, 51]. This is an important finding because chronic kidney disease is present in one-third of patients with heart failure and is associated with increased mortality [52]. Preventing progression of chronic kidney disease may be beneficial for patients with heart failure. The aforementioned meta-analysis by Zannad et al. reported similar efficacy of SGLT2 inhibitors on cardiovascular mortality and hospitalisation for heart failure in patients with HFrEF with an estimated glomerular filtration rate (eGFR) ≥ 60 compared to < 60 ml/min per 1.73 m^2^ [47]. A post hoc analysis from the CANVAS Program that specifically investigated cardiovascular outcomes in diabetic patients with chronic kidney disease demonstrated a greater absolute risk reduction in cardiovascular mortality and hospitalisation for heart failure in those patients with worse kidney function [53].

Common side effects of SGLT2 inhibitors include genital infections and volume depletion. Rarely, euglycaemic ketoacidosis may develop. Patients using sulfonylurea derivates or insulin are more prone to this latter complication and may also develop hypoglycaemia. One study (i.e. the CANVAS Program) reported a higher risk of amputation [40]. However, this finding has not been reported in any other study on SGLT2 inhibitors. In both DAPA-HF and EMPEROR-Reduced, the risk of volume depletion was the same in patients treated with SGLT2 inhibitors and those receiving placebo [45, 46].

Indications for prescribing SGLT2 inhibitors and points of concern in patients with HFrEF are shown in Box 1.

### Proposed working mechanisms of SGLT2 inhibitors

**Fig. 1:**
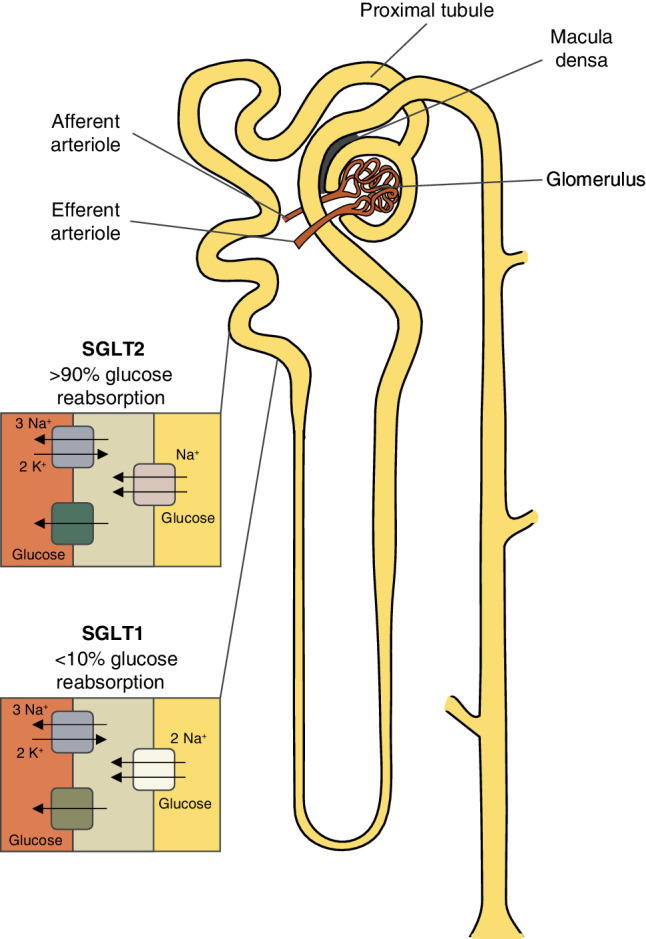
Mechanism of sodium-glucose cotransporter‑2 (*SGLT2*) inhibition. SGLT2 is a sodium-glucose cotransporter located in the proximal tubule and is responsible for > 90% of glomerular filtrate glucose reabsorption. SGLT1, another sodium-glucose cotransporter, is located more distally in the proximal tubule and reabsorbs the remaining glucose. Reabsorbed glucose exits the tubule cell basolaterally through glucose transporters GLUT2 and GLUT1, respectively. SGLT2 inhibitors impede only 50–60% of glucose reabsorption. This incomplete inhibition of glucose reabsorption is caused by a compensatory increase in SGLT1 activity and prevents the development of hypoglycaemia. Copyright © 2007 Yosi I. Adapted from https://commons.wikimedia.org/wiki/File:Nephron.svg distributed under the terms of the Creative Commons Attribution 3.0 Unported License (https://creativecommons.org/licenses/by/3.0/deed.en)

The data above indicate that SGLT2 inhibitors have beneficial cardiorenal effects. The rapidity of onset of these effects suggests a working mechanism independent of glycaemic control. Although the exact working mechanisms of SGLT2 inhibitors are unknown, osmotic diuresis due to glycosuria and concomitant natriuresis seem to be pivotal (Fig. 1; [54]). SGLT2 inhibitors impede the function of sodium-glucose cotransporter‑2, which is located in the proximal tubule of the kidney. The resultant diuresis decreases congestion. Natriuresis also increases the sodium concentration of the glomerular filtrate, which is sensed by the macula densa in the juxtaglomerular apparatus. Due to tubuloglomerular feedback, this results in glomerular afferent arteriolar vasoconstriction. Glomerular afferent arteriolar vasoconstriction reduces hyperfiltration, resulting in nephroprotection with ensuing long-term cardiorenal benefit. Glomerular afferent arteriolar vasoconstriction causes a temporary decline in eGFR.

Other beneficial effects of SGLT2 inhibitors include glycosuria-induced weight loss and a lower production of free radicals and pro-inflammatory cytokines due to reduced mitochondrial dysfunction and ensuing oxidative stress [54]. The resultant decline in inflammation is proposed to prevent fibrosis-induced diastolic and systolic heart failure and may be an appealing mechanism by which SGLT2 inhibitors could exert advantageous effects in patients with HFpEF (i.e. a condition characterised and exaggerated by a pro-inflammatory state) [55]. Favourable effects on endothelial function, as well as cardiomyocyte calcium handling and ketone utilisation, may also be involved in SGLT2 inhibitor-induced cardioprotection in patients with HFrEF and HFpEF [56].

## The effects of antidiabetic agents on heart failure: is it time to update the guidelines?

In the 2016 European Society of Cardiology (ESC) guidelines for the diagnosis and treatment of acute and chronic heart failure, pharmacotherapy with angiotensin-converting-enzyme inhibitors, beta-blockers and mineralocorticoid receptor antagonists is recommended for patients with HFrEF; for patients with HFpEF, diuretics are recommended for congestion. Antidiabetic agents have no important role [57].

**Fig. 2:**
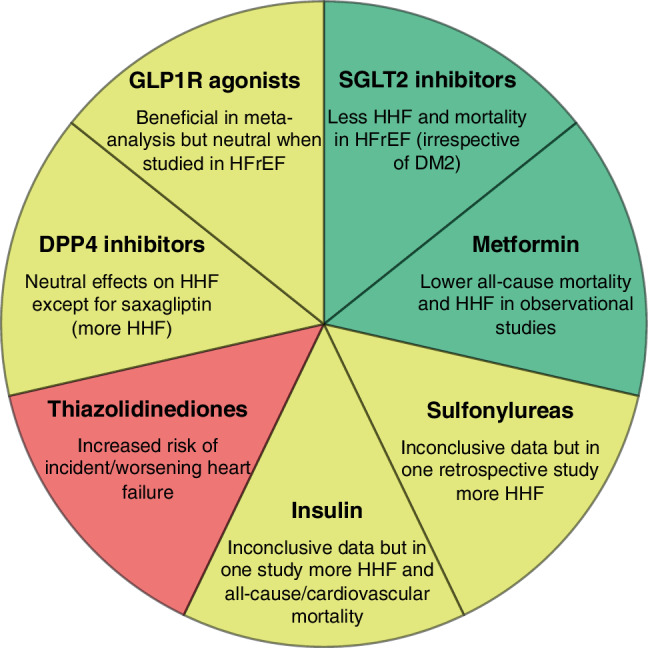
Summary of the effects of antidiabetic agents on heart failure. *ADHF* acute decompensated heart failure, *DM2* diabetes mellitus type 2, *DPP4* dipeptidyl peptidase‑4, *GLP1R* glucagon-like peptide‑1 receptor, *HHF* heart failure hospitalisation, *HFrEF* heart failure with reduced ejection fraction, *SGLT2* sodium-glucose cotransporter‑2

Based on our review (Fig. 2, Box 2) and a recent study by Vaduganathan et al. [58], we have a strong feeling that the ESC guidelines for the diagnosis and treatment of acute and chronic heart failure will be updated shortly. We believe that the evidence is strong enough to recommend SGLT2 inhibitors in patients with HFrEF in addition to angiotensin-neprilysin inhibitors, mineralocorticoid receptor antagonists and beta-blockers. For HFpEF (and HFmrEF), further data are needed before similar recommendations can be made. On 5 November 2020, dapagliflozin was the first SGLT2 inhibitor to be approved for the treatment of HFrEF in diabetic and non-diabetic patients in Europe [59].

## Conclusions and future perspectives

Until recently, the effects of antidiabetic agents on heart failure were largely unknown. This changed after an observed increased risk of heart failure and ischaemic heart disease associated with thiazolidinediones that prompted the requirement for cardiovascular outcome trials for new glucose-lowering drugs. Subsequently, three classes of antidiabetic agents became available (i.e. DPP4 inhibitors, GLP1R agonists and SGLT2 inhibitors). Of these drug classes, only SGLT2 inhibitors significantly and consistently lower the risk of heart failure in patients with diabetes mellitus type 2. Recent trials demonstrated that SGLT2 inhibitors also protect against worsening heart failure in non-diabetic patients with HFrEF. We believe that current evidence is strong enough to recommend SGLT2 inhibitors in both diabetic and non-diabetic patients with HFrEF. Due to the recent approval of dapagliflozin for the treatment of HFrEF in Europe, SGLT2 inhibitors are likely to be reimbursed by health insurance agencies in the near future. Ongoing studies, including EMPEROR-Preserved and DELIVER (Dapagliflozin Evaluation to Improve the Lives of Patients with Preserved Ejection Heart Failure) will hopefully replicate the promising findings of SGLT2 inhibition in diabetic and non-diabetic patients with HFpEF.
